# How many suffice? A computational framework for sizing sentinel surveillance networks

**DOI:** 10.1186/1476-072X-12-56

**Published:** 2013-12-09

**Authors:** Geoffrey Fairchild, Philip M Polgreen, Eric Foster, Gerard Rushton, Alberto M Segre

**Affiliations:** 1 Department of Computer Science, University of Iowa, Iowa City, Iowa, USA; 2 Department of Internal Medicine, University of Iowa, Iowa City, Iowa, USA; 3 Department of Biostatistics, University of Iowa, Iowa City, Iowa, USA; 4 Department of Geography, University of Iowa, Iowa City, Iowa, USA

**Keywords:** Influenza, Outbreak intensity, Outbreak timing, Disease surveillance, Maximal coverage model, K-median model, Huff model, Harmony search, Medicaid, Simulation

## Abstract

**Background:**

Data from surveillance networks help epidemiologists and public health officials detect emerging diseases, conduct outbreak investigations, manage epidemics, and better understand the mechanics of a particular disease. Surveillance networks are used to determine *outbreak intensity* (i.e., disease burden) and *outbreak timing* (i.e., the start, peak, and end of the epidemic), as well as *outbreak location*. Networks can be tuned to preferentially perform these tasks. Given that resources are limited, careful site selection can save costs while minimizing performance loss.

**Methods:**

We study three different site placement algorithms: two algorithms based on the maximal coverage model and one based on the K-median model. The maximal coverage model chooses sites that maximize the total number of people within a specified distance of a site. The K-median model minimizes the sum of the distances from each individual to the individual’s nearest site. Using a ground truth dataset consisting of two million de-identified Medicaid billing records representing eight complete influenza seasons and an evaluation function based on the Huff spatial interaction model, we empirically compare networks against the existing Iowa Department of Public Health influenza-like illness network by simulating the spread of influenza across the state of Iowa.

**Results:**

We show that it is possible to design a network that achieves outbreak intensity performance identical to the status quo network using two fewer sites. We also show that if outbreak timing detection is of primary interest, it is actually possible to create a network that matches the existing network’s performance using 59% fewer sites.

**Conclusions:**

By simulating the spread of influenza across the state of Iowa, we show that our methods are capable of designing networks that perform better than the status quo in terms of both outbreak intensity and timing. Additionally, our results suggest that network size may only play a minimal role in outbreak timing detection. Finally, we show that it may be possible to reduce the size of a surveillance system without affecting the quality of surveillance information produced.

## Background

Although facilities location algorithms were originally used to help firms decide where to build new retail outlets or distribution centers [1], these algorithms have also been used for decades to help allocate healthcare resources. In the United States (U.S.), for example, the Emergency Medical Services (EMS) Act of 1973 required that 95% of service requests had to be served within 30 minutes in a rural area and within 10 minutes in an urban area [2]. More recently, investigators have studied how to locate EMS facilities to aid in large-scale emergencies such as earthquakes or terrorist attacks [3]. In addition to improving responses to healthcare problems, facilities location algorithms have been used to place preventive healthcare services [4] and also to design healthcare systems in developing countries [5]. In previous work, we have shown how to apply facilities location algorithms to design disease surveillance networks [6] and primary stroke center networks [7].

We focus on outpatient influenza surveillance in this paper. The Centers for Disease Control and Prevention (CDC) currently collects different types of infleunza-related information [8]. Although these different systems (Table 1) are in some sense complementary, they were not originally developed to optimize detection of influenza cases in any systematic way (i.e., using an explicit optimization criterion, such as maximizing population coverage or minimizing average distance to population elements). Indeed, these systems were in many cases “networks of convenience”.

**Table 1:** The five categories of ILI surveillance used by the CDC

**Category**	**Description**
Viral surveillance	Approximately 85 U.S. World Health Organization (WHO) Collaborating Laboratories and 60 National Respiratory and Enteric Virus Surveillance System (NREVSS) laboratories located throughout the United States participate in virologic surveillance for influenza.
Outpatient illness surveillance	Information on patient visits to health care providers for influenza-like illness is collected through the U.S. Outpatient Influenza-like Illness Surveillance Network (ILINet). ILINet consists of more than 2,700 outpatient healthcare providers in all 50 states, the District of Columbia and the U.S. Virgin Islands reporting more than 30 million patient visits each year.
Mortality surveillance	122 cities across the United States report the total number of death certificates and the number for which pneumonia or influenza was listed as the underlying or contributing cause of death. Additionally, all influenza-associated deaths in children (age < 18) are reported.
Hospitalization surveillance	Laboratory confirmed influenza-associated hospitalizations in children and adults are monitored through the Influenza Hospitalization Surveillance Network (FluSurv-NET).
Summary of the geographic spread of influenza	State health departments report the estimated level of geographic spread of influenza activity in their states each week spread of influenza through the State and Territorial Epidemiologists Reports.

Surveillance network design has recently been improved using a data-driven approach, incorporating weekly statewide data, hospitalization data, and Google Flu Trends data [9]. Although such methods may provide for better networks in certain instances, many large and populous regions of the world in critical need of surveillance lack the requisite data for such analysis (e.g., poor/untrustworthy records, lack of reasonable influenza activity estimates, lack of Google Flu Trends data in India, China, and all of Africa). Additionally, Google Flu Trends does not track influenza activity perfectly and can differ dramatically from CDC data [10]. Thus, more traditional approaches based on facilities location algorithms that require only population data are still the method of choice for surveillance network design in many regions of the world.

Surveillance networks are used to determine not just *outbreak location*, but also *outbreak intensity* (i.e., disease burden) and *outbreak timing* (i.e., the start, peak, and end of the epidemic). Using networks to detect these factors of disease spread is not new; however, to our knowledge, no other study has examined the implications of designing networks that are tuned to preferentially perform one of these three tasks. Clearly, if one were primarily interested in outbreak intensity or fine-grained outbreak location information, one would want to incorporate as many sites as possible. But given that resources are inevitably limited, careful site selection can save costs while minimizing performance loss; knowing the primary detection task is an important first step in designing more efficient and/or effective networks.

In this paper, we examine site placement for an influenza-like illness (ILI) sentinel surveillance network in the state of Iowa. Iowa is a state in the U.S., roughly 310 miles by 199 miles (500 kilometers by 320 kilometers) in area, populated by approximately three million people. In Iowa, ILI is the major form of outpatient surveillance for influenza activity. ILI is a collection of symptoms that indicate a possible influenza infection (e.g., cough, fever, sore throat). Only laboratory tests can confirm actual influenza. The Iowa Department of Public Health (IDPH) maintained 19 ILI sentinel sites in 2007, comprised of primary care facilities and test laboratories selected strictly on a volunteer basis. We analyze and compare several algorithmic surveillance site placement techniques using Iowa as a test environment, specifically in terms of detecting outbreak intensity and outbreak timing. We examine the proportion of cases detected by the different placement methods under explicit probabilistic detection assumptions. We compare these results against the number of cases that would have been detected by the 2007 IDPH network under identical assumptions. We then use statistical correlation as a means to study outbreak timing. We demonstrate how we can dramatically reduce the size of the surveillance network while still successfully detecting the start, peak, and end of the outbreak.

## Methods

### Online tool

**Figure 1:**
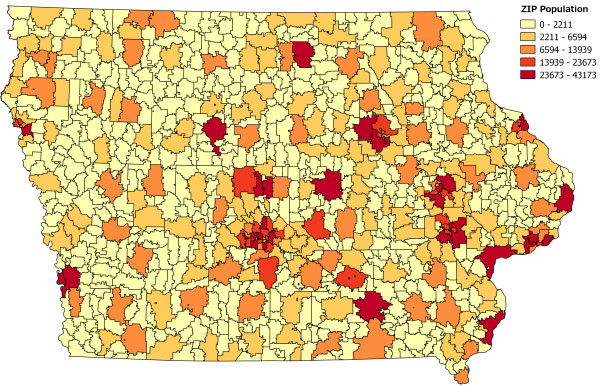
**Map showing population distribution in Iowa by ZIP code.** All 935 ZIP codes in Iowa are shaded by population. Darker colors indicate larger populations while lighter colors indicate smaller populations.

We have developed a web-based calculator that provides a simple user interface for public health officials to determine the best site placement for every state in the U.S. [11] This web application takes as input a list of possible candidate site locations (by ZIP code — there are 935 in Iowa) and, if the user is extending an existing network, a list of any preselected site locations. The user chooses an algorithm and provides any additional parameters specific to the algorithm as well as the total number of sites required. The application then selects a set of sites and overlays the results on a map. Population coverage statistics are also shown. The calculator is capable of designing networks in every state in the U.S. and currently uses 2010 U.S. Census population data. Iowa population distribution by ZIP code is presented in Figure 1.

The methods in this paper operate at the ZIP code level; each surveillance site is represented by the ZIP code in which it resides. Because the ZIP code is an integral part of a site’s address, we can determine the location (i.e., latitude and longitude), and also population, without geocoding the address; we simply consult a look-up table. More fine-grained population data may certainly be used (e.g., block- or tract-level), but addresses must be geocoded in those cases to determine location and population. Our abstraction does not preclude network design in the case where multiple sites are located in the same ZIP code.

### Algorithms

The web-based calculator supports three different network design algorithms: two algorithms based on the maximal coverage model and one based on the K-median facilities location model.

#### Maximal coverage model

The maximal coverage model (MCM) considers each site as having a fixed coverage radius. For example, given that surveillance sites are typically primary care facilities, it may be reasonable to assume that a site may serve patients who live within a 30-minute driving radius of the site (indeed, this is the radius of coverage we use in our simulations). The resulting optimization problem can be stated informally as follows: given a geographic population distribution and a radius of coverage for each site, we wish to choose the sites that maximize the total number of people within the specified distance of a site [12]. Because the problem is non-deterministic polynomial-time hard (NP-hard) to solve exactly (i.e., it is typically infeasible to compute the optimal solution), we instead implement a greedy approximation algorithm that provides a $\left(1-\frac{1}{e}\right)$-approximation of the optimal solution [13]. This approximation algorithm guarantees a rapid solution that is “close enough” to optimal for use in practice.

Note that the standard MCM formulation places no restrictions on the number of cases a site can serve (or in this case, detect). In the real world, however, surveillance sites cannot detect an infinite number of cases, as each site will have some established natural limit, for example, in terms of the number of patients it can serve. Such site capacity constraints are explicitly modeled in the capacitated MCM formulation where each site is endowed with some intrinsic integer capacity. Each person inside the radius of a site *S*_*i*_ is then uniquely counted against that site’s capacity. Once a site’s capacity is exhausted, it may become appropriate to place another site *S*_*j*_ near *S*_*i*_ notwithstanding overlapping site radii. For example, using the standard non-capacitated MCM formulation, sites are preferentially placed in very dense urban areas, often with several hundred thousand people within a single site’s coverage radius. The capacitated model would instead deploy multiple surveillance sites to high density locations in order to account for each site’s intrinsically limited surveillance capacity.

**Figure 2:**
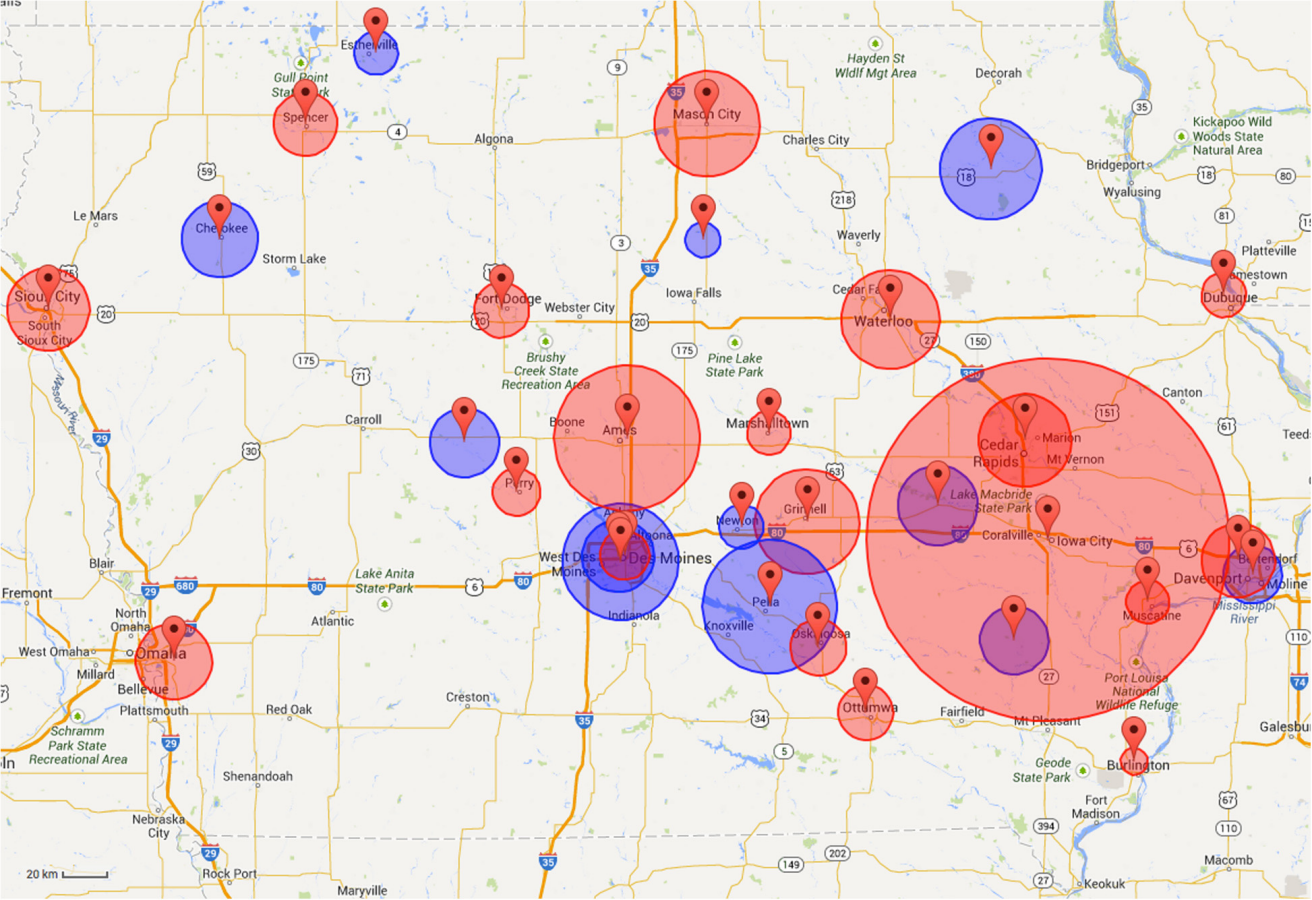
**Map comparing 19 existing sites against 19 sites chosen using MCM-NC.** The 19 existing sites (blue circles) and 19 sites calculated using the MCM-NC (red circles) are shown together. Circles around each marker indicate the average driving distance between patient homes and provider location in the Medicaid dataset. The large red circle around Iowa City represents the University of Iowa Hospitals and Clinics; the average driving distance is 45.47 miles (73.18 kilometers). The MCM-NC tends to choose sites in the more densely populated regions in Iowa. These sites often contain more reputable hospitals and clinics, and as a result, many people are willing to drive further distances to be seen at these locations. The existing network neglects certain populous regions of Iowa (such as Council Bluffs near Omaha) while potentially over-covering other regions (such as Des Moines). Although the Medicaid dataset is used to display average driving distances in this figure, recall that only population data are used to select sites for a network.

Figure 2 shows how 19 sites chosen using the non-capacitated MCM compare against the 19 sites used by the IDPH.

#### K-median model

The K-median model (sometimes also referred to as the P-median model, as in [14]) minimizes the sum of the distances from each individual to their nearest site (a more formal specification is found in [15]). Like the maximal coverage problem, the K-median problem is also NP-hard [16], so an approximation algorithm is once again in order. Here, we use a simple greedy algorithm, although there are more complicated approximation algorithms that can generate slightly better solutions (e.g., [14,17]).

Note that there is a fundamental difference between the maximal coverage model and the K-median model: the K-median model has no explicit notion of population coverage; hence no radius of coverage is involved. By definition, every person in the selected geography is “covered”, although the “quality” of his or her coverage (in terms of travel distance) will vary. For this reason, our web-based calculator always claims 100% coverage when sites are placed using the K-median model.

### Validation

We can evaluate these different methods empirically by simulating the spread of influenza across the state of Iowa and calculating the probability of each case being detected by any surveillance site. Because our simulations are based on a historical record of actual influenza-related cases, we can make meaningful comparisons between the performance of algorithmically-derived surveillance networks and the existing IDPH network.

#### Medicaid dataset

**Figure 3:**
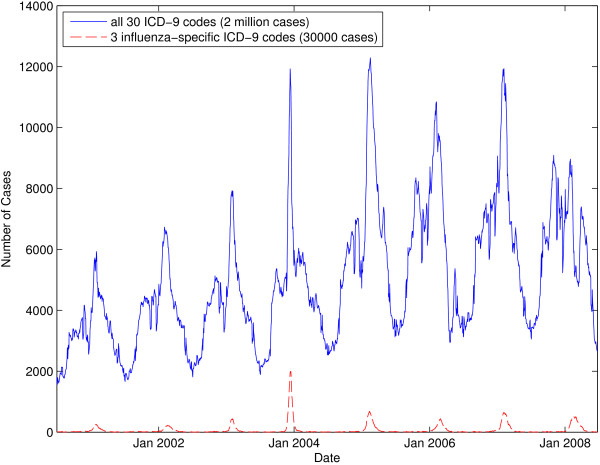
**Seven-day moving average graph showing the number of cases present in the Medicaid dataset over time.** The top line aggregates cases for all 30 ICD-9 codes while the bottom line aggregates cases for three influenza-specific ICD-9 codes (i.e., 487.x). Note that, when using all 30 ICD-9 codes, the case count never goes to zero.

We use a dataset consisting of two million de-identified Medicaid billing records representing eight complete influenza seasons from July 2000 to June 2008. Medicaid is a U.S. federal health insurance program for people and families with low incomes. These records comprise *all* of the Iowa Medicaid records from this time period that contain any one of 30 pre-specified ICD-9 codes that have been previously associated with influenza [18]. Note that we use ICD-9-coded data as a proxy measure for influenza activity because laboratory-based influenza were not available for the state of Iowa. A look at a seven-day moving average graph of the dataset in Figure 3 clearly shows the well-established seasonal influenza peak that occurs each winter [19].

Each record consists of an anonymous unique patient identifier, the ICD-9 diagnosis billing code, the date the case was recorded, the claim type (I — inpatient, O — outpatient, and M — medical), the patient ZIP code, age, gender, and provider ZIP code (Table 2). The dataset is very complete; of the two million total entries, only 2500 entries are dropped due to an erroneous or missing field (e.g., a patient ZIP code of 99999). A second influenza-specific subset of the original data can be defined by selecting only three of the original 30 ICD-9 codes that diagnose laboratory-verified influenza (i.e., 487 — influenza, 487.1 — influenza with other respiratory manifestations, and 487.8 — influenza with other manifestations). These three ICD-9 codes constitute approximately 30,000 entries, or about 4,000 per year. When all 30 ICD-9 codes are considered, the disease seems to never disappear (Figure 3); even during the summer, there are several thousand cases. This might be attributed to the fact that many of the 30 ICD-9 codes present in our expanded dataset include codes that represent diseases and symptoms seen year-round (e.g., cough and acute nasopharyngitis).

**Table 2:** Ten sample entries in the Medicaid billing dataset

**id**	**ICD-9**	**Date**	**Claimtype**	**Patient_zip**	**Age**	**Gender**	**Provider_zip**
2421392	466	12/5/2005	O	50315	43	F	50314
2421392	465.9	1/23/2006	O	50315	43	F	50314
2421392	465.9	2/2/2006	O	50315	43	F	50314
2421392	465.9	3/9/2006	O	50315	43	F	50314
2421392	465.9	11/7/2006	O	50315	44	F	50314
1406011	780.6	11/30/2000	O	50316	37	F	50316
1979061	462	5/16/2001	M	50309	59	F	50315
425531	466	2/2/2004	M	50317	32	F	50316
425531	466	2/12/2004	M	50317	32	F	50313
425531	465.9	8/11/2004	M	50317	32	F	50313

The current diagnosis billing code standard is ICD-10, which provides for more diagnostic granularity than ICD-9. Although our data do not use this new standard, no significant changes would need to be made to the methods used in this paper for validation; only careful selection of ICD-10 codes that correspond to cases of interest is required.

#### Simulation

We treat the Medicaid dataset as a proxy of the record of all ILI cases that occurred in Iowa between 2000 and 2008. The probability of case detection is determined by the Huff model, a probabilistic model often used in geography literature to analyze and understand aggregate consumer behavior [20]. Here, we use the Huff model to determine where people might seek care based on distance to the provider and the provider’s perceived “attractiveness”. More formally, the probability *H*_*ij*_ that case *i* is detected by surveillance site *j* is given by

(1) $$H_{\text{ij}}=\frac{A_{j}^{\alpha }D_{\text{ij}}^{-\beta }}{\overset{n}{\underset{j=1}{\sum }}A_{j}^{\alpha }D_{\text{ij}}^{-\beta }},$$

where *A*_*j*_ is the attractiveness of site *j*, *D*_*ij*_ is the distance from case *i* to site *j*, *α* is the attractiveness enhancement parameter, *β* is the distance decay parameter, and *n* is the total number of surveillance sites.

We use the Huff model because it gives us a way of balancing the “attractiveness” of a site against the distance a patient may be from the site. Although we could use the great-circle distance formula (i.e., geodesic distance on the surface of a sphere) to approximate road distance [21], we instead created a driving distance matrix using Microsoft’s Bing Maps API so that our measurements of travel time are as accurate as possible. *D*_*ij*_ is measured as driving distance in miles.

The challenge of properly setting appropriate values for the attractiveness, attractiveness enhancement parameter, and distance decay parameter remains. One solution, and the one adopted in this work, is to estimate the attractiveness of a site from the number of cases seen at that site in the Medicaid dataset. Since we have a comprehensive set of Medicaid cases on which we use the Huff model, we can fit appropriate values of *α* and *β* from the dataset. Although a number of parameter estimation methods have been proposed (e.g., [22-26]), we present a method that uses a metaheuristic global optimization algorithm called harmony search (HS) [27] to determine the two parameters. HS has been applied to a variety of problems, including other parameter estimation problems, and it often outperforms other commonly used search algorithms, such as simulated annealing, tabu search, and evolutionary algorithms (e.g., [28-35]). We treat our parameter estimation problem as a maximization problem, where the goal is to select values of *α* and *β* that produce the maximal average number of Medicaid cases “correctly” located using the Huff model; a case is “correctly” located if a number selected at random in the range [0,1) is less than the Huff probability, *H*_*ij*_. Case count is averaged over 50 replicates.

We use an open source Python implementation of HS called pyHarmonySearch [36]. *α* and *β* are both allowed to vary in the range (0, 20]. We set max_imp to 100, hms to 20, hmcr to 0.75, par to 0.5, and mpap to 0.25. We ran a total of 20 HS iterations. For the full dataset, the best solution gave us a fitness of 1,032,762.2 cases correctly detected (out of two million total cases) with *α* = 17.998 and *β* = 19.769. For the influenza-specific dataset, the best solution had a fitness of 15,141.14 cases (out of 30,000 total cases) with *α* = 19.114 and *β* = 19.479.

## Results

We simulate influenza spread considering both the entire dataset and the influenza-specific dataset. Because our simulations are stochastic, results are produced by averaging over 50 replicates. Placement algorithms design networks by selecting sites from an IDPH-provided set of 117 candidate sites spread across the state of Iowa. In addition to the MCM and K-median location-allocation models, our analysis considers surveillance networks designed by selecting sites uniformly at random. Results are reported for each network size by averaging over 50 randomly generated networks.

### Outbreak intensity

**Figure 4:**
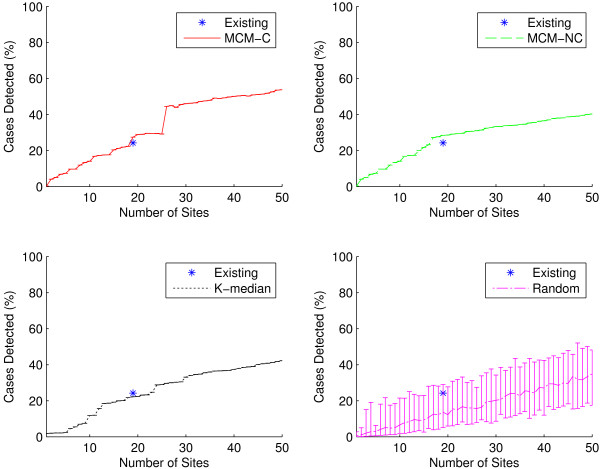
**Outbreak intensity as a function of network size for all ICD-9 codes.** Outbreak intensity is shown for the existing sites (shown as a single data point at *n* = 19) as well as for sites generated using the two MCM variants, K-median model, and randomly selected sites considering all 30 ICD-9 codes. MCM-C is capacitated MCM, and MCM-NC is non-capacitated MCM. Random results at each network size were computed by selecting 50 networks uniformly at random. In all cases, because our simulations are stochastic, results were computed using 50 replicates. Graphs show average results as well as minimum/maximum error bars. In general, the two MCM variants perform best, with the K-median model trailing closely. The two MCM models outperform the existing sites. The existing sites detect approximately 24.2*%* (±0.02*%*) of all cases, while it takes only 17 sites for the MCM-NC to accomplish the same level of outbreak intensity detection. At *n* = 19, MCM-NC detects 28.5*%* (±0.02*%*) of all cases. As the number of sites grows beyond 20 sites, the capacitated MCM better detects outbreak intensity.

**Figure 5:**
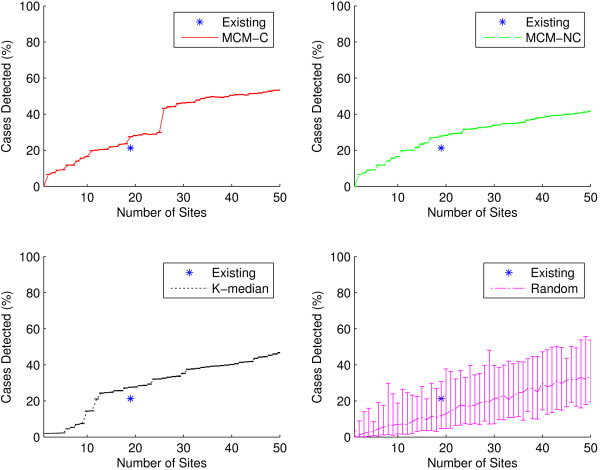
**Outbreak intensity as a function of network size for the influenza-specific dataset.** Outbreak intensity is shown for the existing sites (shown as a single data point at *n* = 19) as well as for sites generated using the two MCM variants, K-median model, and randomly selected sites for the three influenza-specific ICD-9 codes. Here, all three algorithmic variants outperform the existing sites. The existing sites detect approximately 21.2*%* (±0.1*%*) of all cases. It takes only 12 sites for the K-median to accomplish the same level of outbreak intensity detection. At *n* = 19, MCM-NC detects 27.9*%* (±0.2*%*) of all cases.

One way of comparing the quality of two different surveillance networks is to compare the accuracy of their respective measures of outbreak intensity: here the percentage of cases correctly detected by each network using the Huff model. In each graph, the performance of the existing IDPH-selected sites is shown as a single data point at *n* = 19. As seen in Figures 4 and 5, sites generated by the capacitated and non-capacitated MCM (MCM-C and MCM-NC, respectively) tend to perform best, followed closely by the K-median model. Performance improves as network size grows. Unsurprisingly, selecting sites uniformly at random results in worse outbreak intensity detection than preferentially selecting sites.

It seems particularly appropriate to consider the performance of networks of size 19, since this is the number of surveillance sites in the existing IDPH network. At *n* = 19 for the full dataset, we see that all methods, except K-median and random selection, outperform the existing network. As seen in Figure 4, the existing IDPH network detects approximately 24.2*%* (±0.02*%*) of all cases using the full dataset. At *n* = 19, MCM-C detects approximately 27.4*%* (±0.02*%*) of cases, MCM-NC detects approximately 28.5*%* (±0.02*%*), K-median detects approximately 22.2*%* (±0.01*%*), while a random network detects 13.7*%* of cases on average (5.2*%* lower bound, 28.9*%* upper bound). MCM-NC is capable of more efficient detection than the existing network with only 17 sites. For the influenza-specific dataset, as seen in Figure 5, all three algorithmic site placement methods outperform the existing sites. Here, it only takes 12 sites selected using the K-median model to match the outbreak intensity detection of the existing sites. In other words, in the state of Iowa, a network can be designed that detects outbreak intensity as well as the existing network with two fewer sites when considering the full gamut of possible influenza-related ICD-9 codes. However, if we only consider direct diagnoses of influenza, the network can consist of 37% fewer sites. This practically significant result indicates that preferentially selecting sites can yield more efficient surveillance networks with less overhead cost.

### Outbreak timing

In addition to outbreak intensity, a sentinel surveillance network should be able to detect outbreak timing, or the temporal start, peak, and end of a disease season. Intuitively, when attempting to maximize outbreak intensity detection (as well as outbreak location detection), increasing the number of surveillance sites will improve the quality of detection. However, it is not clear that there is an inherent benefit of having more sites when looking at outbreak timing. We would like to explore just how few sites are necessary in order to still accurately detect the timing of a disease season.

A surveillance network will necessarily detect fewer cases than actually occurred among a population; yet, if the surveillance network detects cases temporally in sync with this ground truth, then the disease curve should increase and decrease in proportion with it. We use the Pearson product-moment correlation coefficient (often abbreviated Pearson’s r) to correlate each detected time series with the ground truth dataset in order to quantify outbreak timing detection quality [37]. Correlation coefficients range from -1 to 1. Values above 0.5 and below -0.5 are often interpreted to indicate strong positive and negative correlation, respectively, although these limits are not hard and greatly depend on the context [38]. This method for measuring outbreak timing does not require that we explicitly define the start, peak, or end of a disease season; we simply correlate the simulated disease curves with the ground truth disease curves.

**Figure 6:**
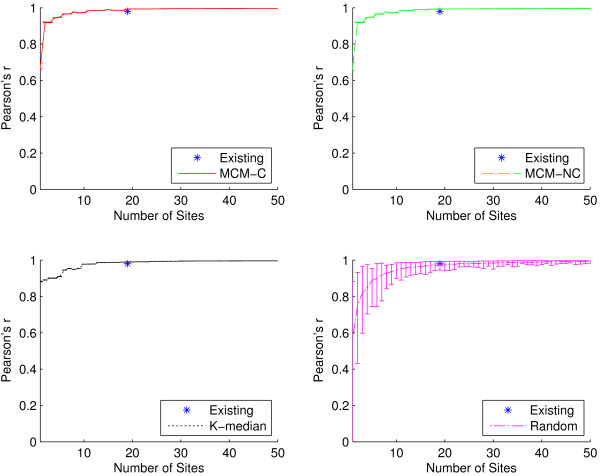
**Outbreak timing as a function of network size for all ICD-9 codes.** Pearson’s r for the existing sites as well as for sites generated using the two MCM variants, K-median model, and randomly selected sites for all 30 ICD-9 codes. Recall that Pearson’s r is used to quantify the quality of outbreak timing detection for a surveillance network. At *n*=19, the average Pearson’s r for the existing sites is 0.98 (±0.0002), while it is 0.99 (±0.0002) for MCM-C, MCM-NC, and K-median, respectively, and 0.97 (±0.02) for random. All algorithmic methods offer site placements that perform extremely well, achieving at least 0.67 correlation for networks with as few as one site. It only requires two sites selected using either MCM variant to achieve correlation of at least 0.9, and only 11 sites are needed to match the performance of the existing network.

**Figure 7:**
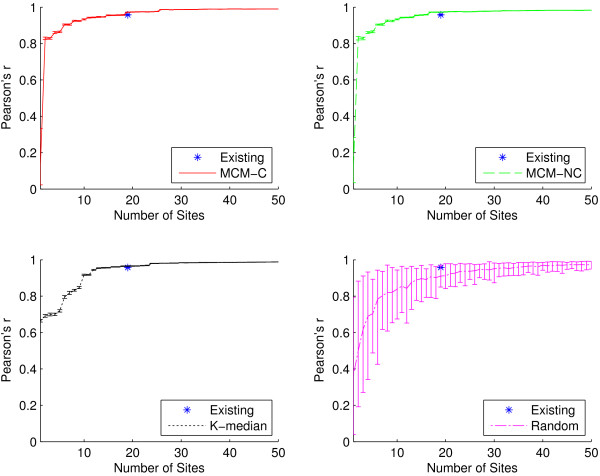
**Outbreak timing as a function of network size for the influenza-specific dataset.** Pearson’s r for the existing sites as well as for sites generated using the two MCM variants, K-median model, and randomly selected sites for the three influenza-specific ICD-9 codes. At *n*=19, the average Pearson’s r for the existing sites is 0.96 (±0.01), while it is 0.97 (±0.001) for all three algorithmic placement methods and 0.91 (±0.06) for randomly placed sites. Compared to Figure 6, there is a very small reduction in outbreak timing detection capabilities when the ICD-9 codes are restricted. Both MCM variants are capable of 0.9 correlation with as few as six sites, and only 14 sites are required to match the performance of the existing network.

Figures 6 and 7 compare the outbreak timing detection capabilities of the algorithmic placement methods and the existing sites using the full dataset and influenza-specific dataset, respectively. In Figure 6, at *n*=19, we see similar outbreak timing performance among all placement methods, with all networks achieving correlation coefficients of at least 0.98 (indicating very strong positive correlation with ground truth). It only takes six algorithmically-placed sites in order to detect outbreak timing at least as well as the existing network, while a network containing only two well-placed sites is capable of achieving a 0.9 correlation coefficient. Even networks with as few as one site are able to achieve correlations of at least 0.67. When the set of ICD-9 codes is restricted to the influenza-specific dataset, as in Figure 7, outbreak timing quality is only slightly reduced. It takes 14 sites to match the performance of the existing network, but it only takes six sites to achieve correlation of at least 0.9. These practically significant findings suggest that it may be possible to drastically reduce the size of a network if the metric of primary interest is outbreak timing detection.

## Conclusions

Disease surveillance is critical in epidemiological studies and in the realm of public health policy. Using a publicly available web-based surveillance site placement calculator and three different algorithmic surveillance site placement methods, we compared the performance of networks generated by the calculator with the volunteer-based network maintained by the IDPH.

The major contribution of this paper is the exploration of two metrics on which a surveillance network can be optimized: *outbreak intensity* and *outbreak timing*. Sites chosen using either MCM variant consistently outperform the baseline IDPH network both in terms of outbreak intensity and timing. Furthermore, we found that preferential selection of sites can yield networks capable of achieving outbreak intensity and timing performance in line with the current IDPH network, requiring, in some cases, only a fraction of the number of sites. We found that, at least in the state of Iowa, the number of sites chosen seems not to matter for outbreak timing detection. This implies that using just a few strategically placed surveillance sites (e.g., Des Moines, Cedar Rapids, Davenport, Sioux City, and Iowa City – the five most populous cities in Iowa) may suffice to reliably and accurately determine the onset, peak, and end of the influenza season in Iowa.

It is important to recognize that although we analyze and compare networks using a dataset of confirmed Medicaid influenza-related cases, network design is accomplished only considering population data. This means that our surveillance network design methods can be used in any location in the world where population data are available.

In practice, surveillance site recruitment, especially in locations where such involvement is voluntary, may prove difficult. This realization opens a new dimension for optimization: cost. Each site brings some inherent cost to the system; the cost may be a monetary value (e.g., incentives), man-hours required for reporting, or some other measure. That is, the real-world optimization problem may actually need to be multi-dimensional. For example, the maximal coverage model may need to be minimal cost, maximal coverage in practice. This direction for future work requires careful consideration when deriving site costs. Additionally, in areas where surveillance site participation is voluntary, a site selected by the methods presented in this paper may decline or hesitate to join the network. The greedy algorithms used here allow for public health officials to rank site importance since, by definition, the most important sites are selected first. This can allow for an adjustment in resource allocation to incentivize important, but unwilling, sites.

In the future, we will look more closely at the problem of selecting the ICD-9 codes worth considering for validation. Here, we only consider two sets of ICD-9 codes: the entire set of all 30 influenza-related ICD-9 codes provided in our Medicaid dataset and an influenza-specific ICD-9 code subset containing only direct diagnoses of influenza (i.e., 487.x ICD-9 codes). One possible approach is to apply machine learning techniques typically used for feature selection to the problem of finding which ICD-9 codes should be used for validation. We will also examine other states exhibiting different population density and geographic characteristics from Iowa, and, eventually, nationwide and worldwide surveillance networks. Ultimately, our goal is to use computational methods to reliably advise public health officials how many surveillance sites suffice and where to place them in order to meet their specific needs.

There are several limitations of our work. First, it it important to recognize that *all* surveillance networks have difficulty making conclusions about uncovered areas. Our methods focus primarily on densely populated regions, so less densely populated regions may be left uncovered. Second, this paper focuses on the state of Iowa in the U.S., which is a relatively simple state geographically and geologically. A more geographically or geologically diverse state such as Colorado with its natural east-west Rocky Mountain division may provide different obstacles in site placement. Third, our placement models ignore demographics, so it is possible the resulting networks are sampling some demographics more than others or possibly missing some demographics altogether. Moreover, the Medicaid data used in our simulations represent a particular demographic of Iowa: people and families with low incomes (these data, however, are complete with respect to that particular demographic). Fourth, all calculations consider the population of a ZIP code to be concentrated at the centroid of that ZIP code. In reality, populations are usually distributed in some fashion across the entire ZIP code region. Additionally, while our simplifying site-as-ZIP code abstraction may be reasonable for less densely populated regions, such as Iowa, it may prove to be problematic in more densely populated regions. A final limitation to our work is that we use administrative data (ICD-9 codes) as a proxy for influenza activity. We would rather use actual ILI data or laboratory-based data, but these data sources were not available across the state.

Our web-based tool can aid public health officials in designing an effective disease surveillance system. We studied two metrics by which a surveillance network may be evaluated: outbreak intensity and outbreak timing. By simulating the spread of influenza across the state of Iowa, we show that the sites our tool selects perform better than the status quo in terms of both metrics. Additionally, we offer new insights that suggest that network size may only play a minimal role in outbreak timing detection. Finally, we show that it may be possible to reduce the size of a surveillance system without affecting the quality of surveillance information the system is able to produce.

## Abbreviations

CDC: Centers for disease control and prevention; EMS: Emergency medical services; HS: Harmony search; IDPH: Iowa Department of Public Health; ILI: Influenza-like illness; MCM: Maximal coverage model; MCM-C: Capacitated maximal coverage model; MCM-NC: Non-capacitated maximal coverage model; NP-hard: Non-deterministic polynomial-time hard; Pearson’s r: Pearson’s product-moment correlation coefficient; U.S.: United States.

## Competing interests

The authors declare that they have no competing interests.

## Authors’ contributions

GF wrote all of the software, drafted the manuscript, and developed the correlation method for quantifying outbreak timing detection. PMP provided medical guidance in understanding influenza’s spread across Iowa as well as the initial research directions based on the maximal coverage model. EF provided statistical guidance and help in understanding and processing data. GR provided geographic guidance and suggested the use of the K-median model as well as the Huff model. AMS helped coordinate project goals and ideas and guided validation efforts. All authors edited the manuscript. All authors read and approved the final manuscript.
